# Metal Organic Frameworks Based Wearable and Point-of-Care Electrochemical Sensors for Healthcare Monitoring

**DOI:** 10.3390/bios14100492

**Published:** 2024-10-10

**Authors:** K Theyagarajan, Young-Joon Kim

**Affiliations:** 1 Department of Electronic Engineering, Gachon University, Seongnam 13120, Republic of Korea; thektr@gachon.ac.kr; 2 Department of Semiconductor Engineering, Gachon University, Seongnam 13120, Republic of Korea

**Keywords:** metal–organic framework, electrochemical biosensor, wearable sensor, continuous monitoring, point-of-care

## Abstract

The modern healthcare system strives to provide patients with more comfortable and less invasive experiences, focusing on noninvasive and painless diagnostic and treatment methods. A key priority is the early diagnosis of life-threatening diseases, which can significantly improve patient outcomes by enabling treatment at earlier stages. While most patients must undergo diagnostic procedures before beginning treatment, many existing methods are invasive, time-consuming, and inconvenient. To address these challenges, electrochemical-based wearable and point-of-care (PoC) sensing devices have emerged, playing a crucial role in the noninvasive, continuous, periodic, and remote monitoring of key biomarkers. Due to their numerous advantages, several wearable and PoC devices have been developed. In this focused review, we explore the advancements in metal–organic frameworks (MOFs)-based wearable and PoC devices. MOFs are porous crystalline materials that are cost-effective, biocompatible, and can be synthesized sustainably on a large scale, making them promising candidates for sensor development. However, research on MOF-based wearable and PoC sensors remains limited, and no comprehensive review has yet to synthesize the existing knowledge in this area. This review aims to fill that gap by emphasizing the design of materials, fabrication methodologies, sensing mechanisms, device construction, and real-world applicability of these sensors. Additionally, we underscore the importance and potential of MOF-based wearable and PoC sensors for advancing healthcare technologies. In conclusion, this review sheds light on the current state of the art, the challenges faced, and the opportunities ahead in MOF-based wearable and PoC sensing technologies.

## 1. Introduction

Due to the widespread prevalence of chronic and infectious diseases, proper healthcare has become one of the most fundamental human needs, alongside food (including air and water) and shelter [1,2]. However, providing adequate healthcare, controlling disease spread, and managing emergencies for a global population of over 8 billion people is extremely challenging, if not impossible, with current technologies [3,4]. This has led to the development of modern healthcare systems equipped with state-of-the-art diagnostic and treatment technologies. These modern healthcare systems focus on early detection of incurable diseases, patient-friendly diagnosis and treatment procedures, remote or continuous monitoring of biologically significant analytes, early prediction of disease development and prognosis, and more [5,6,7]. Furthermore, every individual, at some point in life, from birth to death, will need to undergo some form of diagnosis for proper treatment and care. Most of the current diagnostic procedures are invasive, time-consuming and often require blood samples for various analyses. This process can be painful and sometimes lead to undesirable side effects, such as injuries or infections [8,9,10]. Numerous advanced telemedicine devices have been developed to address these challenges, opening new avenues in the medical field. These telemedicine technologies can integrate chemical transducers with wearable electronics and connect with the internet of things (IoT) to create smart sensors [11,12]. With the advent of flexible and portable electronics, wearable sensors have gained significant attention for real-time monitoring of biomarkers in dynamic bodily fluids. These wearable sensors are noninvasive or minimally invasive, meaning that patients or users do not need to undergo a painful sample collection process. Instead, data can be collected and monitored conveniently without piercing the skin, allowing remote communication with patients and healthcare professionals [13,14].

Compared to various analytical techniques, electrochemical (bio)sensors (ECBS) are among the most promising candidates for several reasons. They are cost-effective, deliver rapid responses, provide good selectivity and sensitivity, require only a small sample volume, can be miniaturized, and offer excellent operational convenience [15,16,17]. Additionally, they can be used for onsite and in situ measurements. Due to these remarkable properties, ECBS are widely employed in detecting and quantifying a broad range of analytes, starting from bio-analytes to environmental pollutants and industrial wastes [18,19,20]. Consequently, ECBS have been developed and utilized to detect and quantify almost every type of analyte. Electrochemical-based wearable and PoC sensors have recently gained popularity due to their superior advantages, such as a small size, portability, rapidity, cost-effectiveness, and applicability in remote sensing and continuous monitoring [21,22]. Designing suitable electrode materials is the most crucial step in the fabrication of ECBSs because it directly influences the performance of the developed sensors [23,24]. Consequently, many researchers and research groups have developed and utilized various materials, molecules, and hybrid structures for the sensitive and selective detection of biologically significant analytes. These include metal nanoparticles, carbon nanostructures, 0D-3D materials, quantum dots, ionic liquids, polymers, metal complexes, metal oxides, organic frameworks, and so forth [25,26,27]. Each material has its own advantages and disadvantages, depending on the intended application. Some materials may be stable in controlled environments but fail to perform in real-world scenarios. Others might perform exceptionally well in practical applications but may not be suitable for long-term use or may have high production costs that outweigh their benefits. Therefore, the ideal sensing material should not only deliver high performance but also be suitable for sustainable bulk production and cost-effective for large-scale applications.

**Figure 1:**
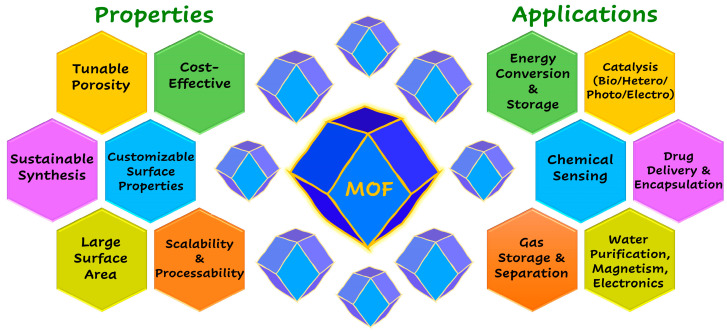
Graphical representation of properties and applications of MOFs.

Metal–organic frameworks (MOFs) are one such candidate, as they can be sustainably synthesized in large quantities at a comparatively lower production cost and can be tailored for various task-specific applications [28,29]. MOFs are porous crystalline materials and inorganic–organic hybrids composed of metal ions/clusters connected to organic linkers in one- or multi-dimensional arrays. They possess various advantageous properties, including a large surface area, tunable pores, controllable morphologies, and intriguing surface characteristics [30,31]. Owing to these merits, MOFs and their derivatives are widely explored in various applications, including energy storage and conversion, gas storage and separation, drug delivery, chemical sensing, catalysis (bio/heterogeneous/photo), biomedicine, and more [32,33,34]. In particular, MOF-based materials have been extensively studied in various electrochemical applications such as electrochemical sensing, electrocatalysis, and electrochemiluminescence [35,36]. Some of the well-known properties and established applications of MOFs are illustrated in Figure 1. Among the multifarious properties of MOFs, some are particularly suited for fabricating modified electrodes and electrochemical sensors. The large active surface area of MOFs enhances the electrode’s effective surface area, facilitating the better adsorption of reactants and improving their electrochemical conversion [37]. Their uniform pore size and volume favor the encapsulation and entrapment of biomolecules and other materials, thereby stabilizing and enhancing their electrochemical and electrocatalytic activities [38]. Moreover, using different synthetic routes, one or more chemical functionalities can be incorporated into the pores or on the surface of MOF structures, making them highly suitable for the selective and sensitive detection of target analytes [39,40,41,42]. Additionally, MOF materials’ cost-effective and sustainable production enables the development of affordable and environmentally friendly sensing devices [43]. Although MOFs offer several advantages for developing ECBSs, certain challenges still exist and must be addressed. These include low electrical conductivity, reduced biocompatibility, and poor stability in aqueous solutions [44,45,46]. Recently, several strategies have been developed and implemented to overcome these difficulties, with many research groups working to improve the conductivity, stability, and biocompatibility of MOFs for better performance in electrochemical sensors.

In recent times, MOF-based ECBSs for sensing biologically, industrially, and environmentally significant analytes have been continuously explored [47,48]. As a result, several interesting reviews have summarized the importance of MOFs and their applicability in electrochemical (bio)sensing [49,50,51,52]. Interestingly, MOF-based wearable and PoC electrochemical sensors for healthcare monitoring represent an emerging technology that only a few research groups have investigated. So far, only two reviews have been published on this topic, but they have not highlighted the core aspects of this area [53,54]. Therefore, we aim to write a focused short review on this essential topic. Instead of summarizing the various properties and applications of MOFs, we specifically focus on MOF-based wearable and PoC sensors developed for healthcare monitoring. This review is organized by analyte, making it highly relevant for researchers in this field and providing an update on current progress. This approach will enable researchers to advance the field by exploring untapped research areas. Furthermore, emphasis has been placed on the opportunities and potential strategies for implementing MOFs and their derivatives in designing and fabricating high-performance wearable and PoC electrochemical sensors.

## 2. MOF Based Wearable and Portable Electrochemical Sensors

In the following section, we have summarized various MOF-based wearable and PoC sensors developed for the electrochemical sensing of biologically and clinically significant analytes. This includes pristine MOFs, MOF hybrids, composites, and their derivative-based sensors. For a better understanding and rational comparison, this section has been categorized analyte-wise.

### 2.1. Cortisol

**Figure 2:**
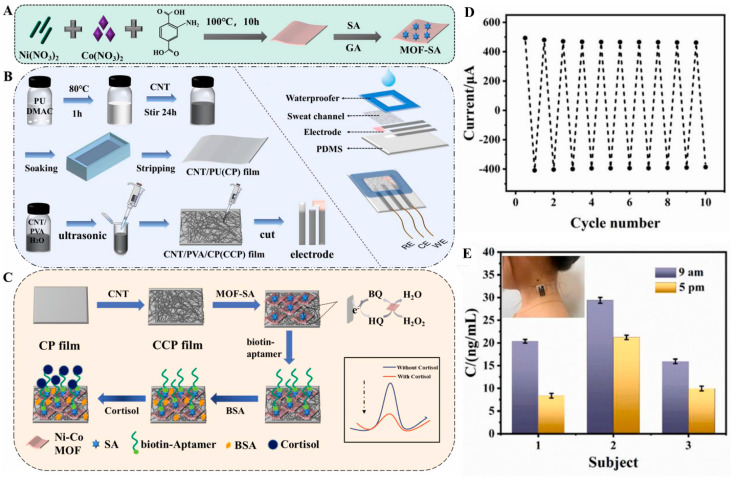
(**A**) Scheme for the synthesis of bimetallic MOF and streptavidin-conjugated MOF. (**B**) Fabrication of flexible and wearable patch sensors. (**C**) Scheme for the fabrication of a cortisol sensor. (**D**) Redox peak current changes in the sensor in the presence of hydroquinone and hydrogen peroxide for 10 cycles. (**E**) Cortisol detection in sweat samples (inset: photograph of a volunteer wearing the patch sensor). Reproduced with permission from [55].

Cortisol is a stress biomarker and plays a significant role in various physiological processes, including the regulation of blood pressure, blood sugar, and metabolism in multiple organs. Cortisol is present in various bodily fluids such as sweat, saliva, urine, blood, and interstitial fluids. Additionally, cortisol production follows a circadian rhythm, with the highest concentration occurring in the morning, gradually decreasing as the day progresses. Therefore, the close monitoring of cortisol levels helps assess the body’s physiological state and prevent adverse effects. Su and colleagues developed a wearable electrochemical aptasensor using bimetallic (Ni-Co) MOF nanosheets decorated with CNT/polyurethane (PU) films for the monitoring of cortisol [55]. At first, a CNT/PU film was formed, followed by streptavidin-conjugated MOF (SA-MOF) deposition to obtain the desired sensor. This biofunctional MOF effectively captures the cortisol aptamer through streptavidin–biotin interaction due to its excellent specific surface area. In the absence of cortisol, the sensor can oxidize hydroquinone by hydrogen peroxide. However, in the presence of cortisol, this catalytic reaction was suppressed due to the formation of an aptamer–cortisol complex. Based on this principle, the sensor quantifies cortisol concentration sensitively and selectively over a linear range of 0.1 to 100 ng mL^−1^, with a limit of detection (LOD) of 32 pg mL^−1^. Furthermore, the sensor was adapted into a wearable patch by assembling it on a polydimethylsiloxane (PDMS) substrate equipped with a sweat collection channel. This flexible patch sensor successfully quantified the cortisol concentrations directly from volunteers’ sweat, demonstrating significant potential for stress monitoring and management. A schematic illustration of material synthesis, sensor fabrication, and its responses are shown in Figure 2.

### 2.2. SARS-CoV-2

**Figure 3:**
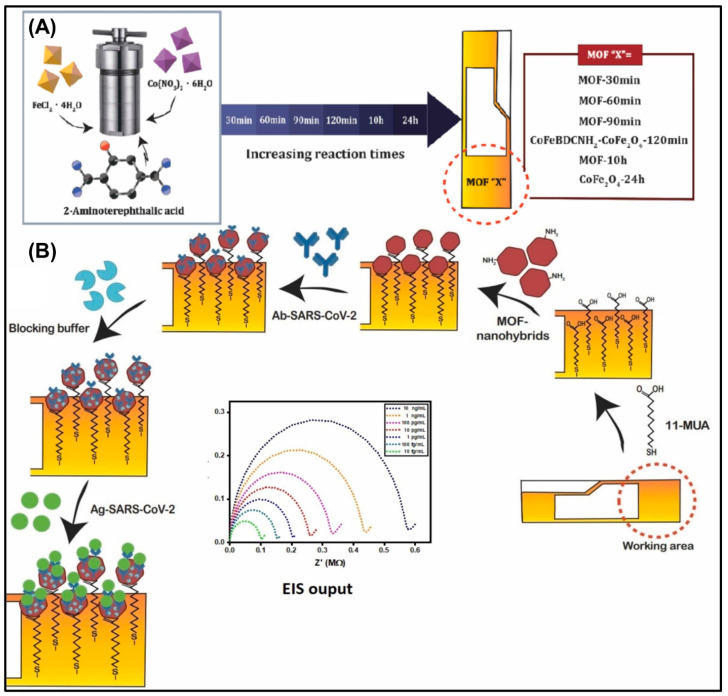
(**A**) Graphical representation for the hydrothermal synthesis of CoFe-MOF-CoFe_2_O_4_ nanohybrids. (**B**) Stepwise fabrication of the SARS-CoV-2 sensor and its electrochemical response. Reproduced with permission from [56].

An MOF nanohybrid-integrated PoC diagnostic device was fabricated for the sensitive and selective detection of the SARS-CoV-2 viral antigen [56]. SARS-CoV-2 is a member of a large family of viruses known as coronaviruses and is responsible for causing respiratory illness that recently led to a pandemic. It can infect both humans and certain animals. The timely detection of SARS-CoV-2 is crucial for preventing the spread of the disease and safeguarding living beings. A nanohybrid consisting of amine-terminated CoFe-MOF combined with CoFe_2_O_4_ spinel ferrites was synthesized hydrothermally and covalently coupled to mercaptoundecanoic acid-functionalized gold chips via EDC/NHS coupling. The resulting chips were then immersed in a SARS-CoV-2 antibody solution to form the desired sensor, as illustrated in Figure 3. The developed sensor successfully detected recombinant SARS-CoV-2 with an LOD of 6.68 and 6.20 fg mL^−1^ in buffer and 10% serum samples, respectively. Furthermore, the sensor was integrated into a handheld device to validate its function in a PoC platform, demonstrating acceptable performance compared to potentiostat results. The sensor’s superior performance was attributed to the effective integration of Co and Fe in the MOF and ferrite structures, which synergistically improved the active surface area, conductivity, and biocompatibility.

### 2.3. Creatinine

Creatinine (CRT) is an important compound found in blood that measures kidney health. It is a by-product of protein metabolism. Healthy kidneys efficiently filter out the excess creatinine, ensuring that only an optimum amount remains in the blood. Thus, measuring creatinine concentration is crucial for assessing kidney health and function. Kalasin et al. developed a lab-on-eyeglasses-based wearable sensor for predicting serum creatinine levels using tear creatinine [57]. The sensor was fabricated using copper oxide NPs hybridized with a copper-containing benzenedicarboxylate MOF (Cu-MOF) bound to graphene oxide–Cu(II). The working electrode (WE) was constructed by dipping a cotton thread in a carbon black solution, followed by electrodeposition with copper oxide NPs. It was then electrochemically coated with Cu-MOF and GO-Cu(II) to form the desired sensor. The reference electrode (RE) was made by coating the cotton thread with carbon black, polyvinyl alcohol, and Ag/AgCl, while the counter electrode (CE) consisted of carbon black and propylene glycol diacetate-coated cotton thread. The developed sensor demonstrated an excellent electrocatalytic detection of CRT in both artificial and human tear samples, exhibiting a linear detection range of 1.6–2400 µM. Using this sensor, a wearable lab-on-eyeglasses fitted with these disposable fabric electrodes was fabricated and utilized for CRT sensing.

### 2.4. Glucose

Glucose is an essential nutrient and key metabolite for human cells. However, blood glucose levels must be within an optimal range, as abnormal levels can lead to serious health problems [58,59]. Diabetes mellitus is a chronic disease caused by hyperglycemia, which is characterized by elevated blood glucose levels over a prolonged period. Periodic or continuous glucose monitoring is essential for the treatment and management of diabetes [60]. Currently, glucose is primarily monitored through blood samples collected invasively from patients. These methods are painful and unpleasant and increase the risk of infection. Interestingly, glucose measurement through wearable sensors is noninvasive or minimally invasive. Moreover, wearable and PoC sensors offer the advantage of continuous and remote monitoring [61,62]. Electrochemical glucose sensing can be performed in both enzymatic and non-enzymatic pathways.

#### 2.4.1. Non-Enzymatic Glucose Sensors

**Figure 4:**
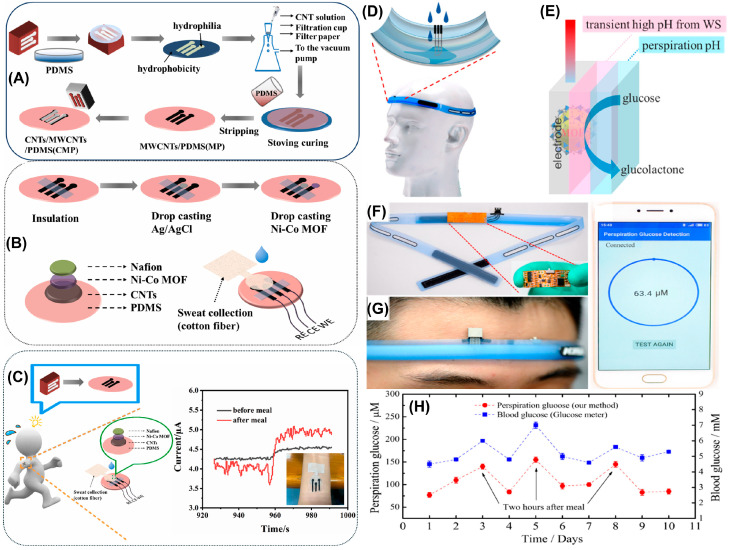
(**A**) Stepwise fabrication of CNT/MWCNT/PDMS and (**B**) fabrication of glucose sensor. (**C**) Current response obtained using the developed sensor for the detection of glucose in volunteers’ sweat. Reproduced with permission from [66]. (**D**) Graphical representation of the fabricated headband. (**E**) Non-enzymatic glucose sensing using the headband. (**F**) Actual photograph of headband integrated with glucose sensor. (**G**) Photograph of a volunteer wearing the headband and perspiration analysis using a smartphone. (**H**) Glucose concentration measured in the perspiration and blood of a human for 10 days. Reproduced with permission from [68].

A high-performance wearable non-enzymatic glucose sensor was developed using 2D bimetallic Ni-Co MOF nanosheets [63]. The sensor was constructed by drop-coating the MOF solution onto the working electrode and allowing it to dry. Subsequently, the sensor was covered with a layer of polyvinyl alcohol (PVA)/KOH gel. This electrode functions both as a micro-supercapacitor and a glucose sensor, making it suitable as a wearable power source and glucose monitoring system. The sensor demonstrated excellent sensitivity for glucose detection and was successfully employed for glucose sensing in sweat samples. The superior performance of this system can be attributed to the synergy between the metal ions. Additionally, the competition between the metal ions leads to the formation of unsaturated metal sites and defects. These defects create more reaction sites and facilitate the entry of glucose molecules for the oxidation process. Furthermore, a wearable biosensor was successfully constructed using this sensor via magnetron sputtering on a polyethylene terephthalate (PET) substrate, which exhibited a sensitivity of 0.31 µA/µM for glucose sensing. Similarly, Shu and colleagues utilized Ni-Co MOF nanosheet-modified wearable sensors for glucose sensing from sweat samples [64]. A stretchable fiber was initially fabricated using RGO and PU via wet spinning technology. The fiber was then coated with a layer of conductive silver, followed by a synthesized Ni-Co MOF to obtain the desired sensor. The reference electrode was fabricated by coating a Ag/AgCl paste over the Ag/RGO/PU electrode, and a platinum wire was used as the auxiliary electrode. All three electrodes were placed on a waterproof bandage and covered with a sweat-absorbent cloth to collect the sweat. This sensor was successfully utilized to continuously monitor sweat glucose in various human subjects, and its reliability was verified against a commercial glucose meter. The sensor offers several advantages, including good mechanical stability, high electrocatalytic activity, acceptable stability, and selectivity for glucose determination. In another work by Yuan et al., a portable sweat-based glucose analysis device was fabricated using Ni-Co MOFs [65]. The device consists of three layers, namely the accelerated diffusion layer, the detection layer, and the hydrophobic layer. The WE and counter electrode (CE) were made by printing a layer of carbon ink onto a nylon film, while the reference electrode (RE) was made by printing silver ink. Finally, the synthesized MOF was coated onto the WE to construct the desired sensor. The diffusion and hydrophobic layers are made with unidirectional polyester polypropylene blended fabrics (honeycomb) with the detection layer inserted between these two layers. The diffusion layer was attached to the skin, where sweat was collected and diffused to the detection layer. The detection layer performs the analysis and transmits a readable signal. This approach allowed the developed sensor to quantify the concentration of glucose present in sweat. Xia and colleagues recently constructed a wearable glucose sensor using a Ni-Co MOF, CNT, and PDMS [66]. Initially, the three-electrode setup was patterned with a PDMS film and then coated with MWCNT and CNT inks to create a CNT/MWCNT/PDMS layer. Subsequently, the synthesized MOF was then drop-coated onto the surface and left to dry. Finally, the working electrode was covered with a layer of Nafion solution to obtain the desired sensor, as shown in Figure 4A,B. Furthermore, a sweat-absorbent cloth was attached to this sensor to collect sweat from the skin for analysis (Figure 4C). Real-time glucose measurements were conducted with volunteers before and after meals, and the developed sensor successfully quantified glucose from the sweat of the volunteers. All the previously discussed sensors commonly utilize Ni-Co MOFs for glucose sensing. The reason for exploring the Ni-Co MOF is owing to their larger surface area, good porosity, ease of synthesis, good mechanical stability even when the sensor is twisted or bent, good biocompatibility, and superior electrocatalytic activity than their monometallic counterparts.

Recently, Rebecca et al. fabricated a self-powered wearable sensor using a ZIF-8/RGO hybrid [67]. Initially, a ZIF-8 MOF and RGO were synthesized separately and mixed via ultrasonication. The resulting mixture was repeatedly washed, centrifuged, and dried to obtain the desired MOF/RGO electrocatalyst. The electroanalytical and catalytic performance of the electrocatalyst for glucose detection was investigated by coating it onto a pencil graphite electrode (PGE). The sensor demonstrated good electrocatalytic activity, stability, and high sensitivity for glucose detection. Using this, a two-electrode wearable patch sensor was developed and utilized for glucose detection from sweat, which showed a strong correlation with standard glucose meters. In another work, a wearable headband was developed for glucose sensing using PdNPs encapsulated with a Co-MOF (ZIF-67) [68]. The Co-MOF was synthesized first, and then PdNPs were encapsulated by an impregnation–reduction process to obtain Pd@Co-MOF. This Pd@Co-MOF was mixed with conductive carbon (CC) ink and printed on a PET film to obtain the WE of the proposed sensor. Similarly, the CE was made by screen-printing CC ink, while the RE was specifically constructed by coating a polymer layer (PVA) containing KCl over a Ag/AgCl electrode. The sensor, on initial assessment, did not exhibit any electrochemical redox reactions; however, after pretreating the sensor at −2.0 V for 20 s, it created a temporary basic environment near the sensor surface, which was sufficient enough to oxidize the glucose present in sweat. A sweatband incorporating this sensor was developed, and its applicability for glucose detection in sweat was investigated, showing satisfactory results when compared with blood glucose measurements (Figure 4D–H). Moreover, the sweatband was designed to transmit real-time data to a smartphone. Similarly, a portable non-enzymatic electrochemical glucose sensor was constructed using a Cu-MOF electrodeposited over a PtNP’s deposited gold electrode (AuE) [69]. The authors constructed three different sensors, namely (1) AuNPs/CuNPs/AuE, (2) NiNPs/CuNPs/AuE, and (3) Cu-MOF/PtNPs/AuE, and compared their electroanalytical properties for glucose determination. The Cu-MOF/PtNPs/AuE exhibited a superior electrocatalytic response compared to others, likely due to the enhanced synergistic effect of the MOF with NPs rather than NP–NP interaction. Furthermore, they have constructed a portable glucose meter using this sensor and compared their performance with a commercial glucose meter, which showed a comparable response.

#### 2.4.2. Enzymatic Glucose Sensors

**Figure 5:**
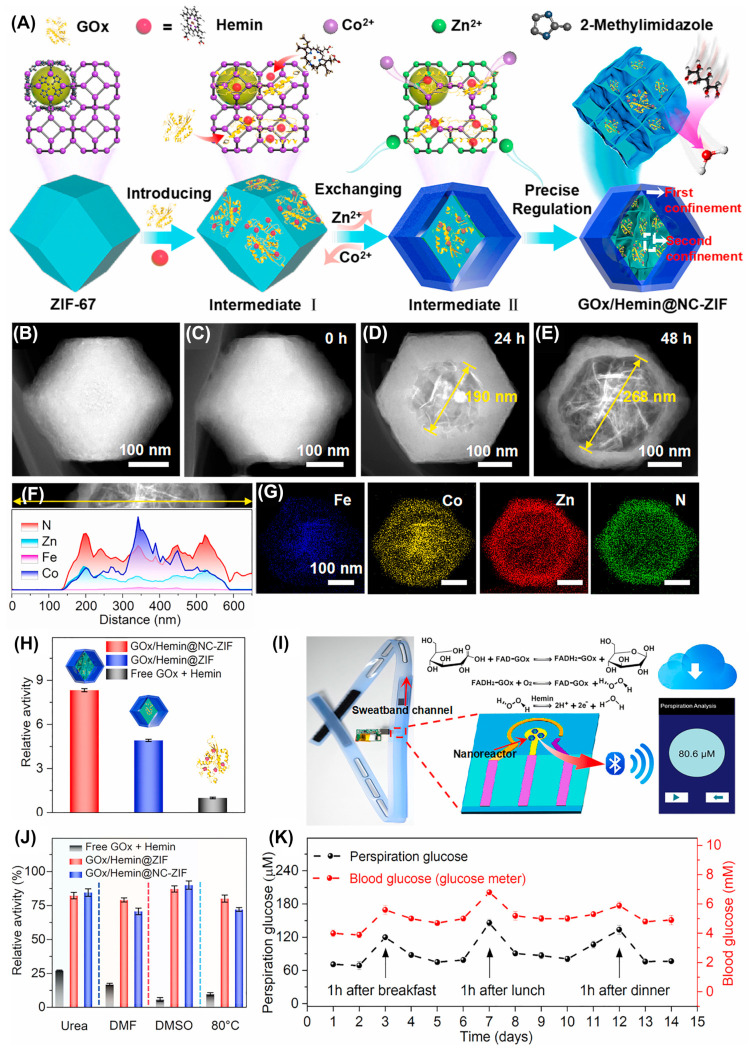
(**A**) Graphical representation for the stepwise synthesis of GOx/Hemin@NC-ZIF. (**B**–**E**) TEM images at different etching times. (**F**) HAADF and its EDS line scan. (**G**) EDS mappings for various elements. (**H**) Relative activities of different electrocatalysts. (**I**) Photograph of wearable sweatband and its catalytic mechanism toward glucose oxidation along with perspiration analysis on a smartphone display. (**J**) Relative activities of different electrocatalysts in various reaction conditions. (**K**) Glucose concentration obtained through perspiration and blood of a human within 14 days. Reproduced with permission from [70].

**Figure 6:**
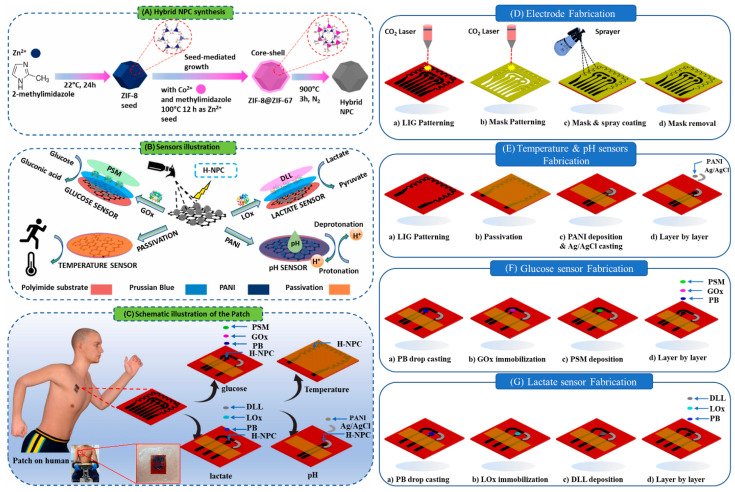
Graphical representation of (**A**) stepwise synthesis of hybridized nanoporous carbon, (**B**) construction of glucose, lactate, temperature, and pH sensors, and (**C**–**G**) stepwise fabrication of the wearable patch sensor and photograph of a volunteer wearing the patch sensor during exercise. Reproduced with permission from [71].

Wang and colleagues developed a nanocage-based ZIF network for the effective encapsulation and stabilization of multiple enzymes and utilized it for the optical and electrochemical sensing of glucose [70]. The sensor consists of nanocages formed by a bimetallic Co-Zn MOF, glucose oxidase (GOx), and Hemin. During the formation of the Co-MOF, GOx and Hemin were added to the reaction mixture. Subsequently, a zinc nitrate solution was added to replace some of the Cu ion centers with Zn ions, yielding the desired electrocatalyst GOx/Hemin@NC-ZIF, as shown in Figure 5A. Using this electrocatalyst, a carbon paste electrode (CPE) was constructed, and its electrochemical properties were explored, which displayed superior activity toward glucose. Furthermore, a wearable sweatband was developed using the designed sensor and utilized for human perspiration analysis, which showed comparable performance to a blood glucose monitor (Figure 5K). In another study, an epidermal patch-based multi-sensor was developed for sensing glucose, lactate, pH, and temperature using hybridized nanoporous carbon (HNPC) [71]. The HNPC was synthesized by annealing the previously prepared core–shell ZIF-8@ZIF-67 MOF crystals, as shown in Figure 6A. The resulting catalyst was spray-coated onto a patterned polyimide film (PIF) and then modified accordingly. The HNPC/PIF was electrodeposited with polyaniline for pH and temperature sensing. For glucose sensing, the HNPC/PIF was further modified with Prussian blue (PB) nanoparticles and GOx and covered with a layer of permselective membrane (PSM) (Figure 6F). For lactate sensing, the sensor was modified with lactate oxidase and then covered with a diffusion-limiting layer composed of dioctyl sebacate, polyvinyl chloride, and tetradodecylammonium- tetrakis (4-chlorophenyl) borate (Figure 6G). The developed sensors demonstrated excellent electrocatalytic activity toward glucose and lactate and as such, a perspiration sensor was constructed. The proposed sensor showed good correlations during the real-time analysis due to temperature and pH corrections. Recently, a touch-based sweat sensor was developed by co-encapsulating enzymes and carbon dots (CDs) within an MOF structure for sensing glucose and lactate [72]. The electrocatalyst was synthesized by encapsulating GOx and arginine-derived CDs in the ZIF-8 nanostructure. The resulting hybrid-zyme was immobilized over a PB-modified carbon-based flexible electrode and then covered with layers of Nafion, chitosan, and sweat-collecting gel to create the proposed sensor. Similarly, lactate oxidase (LOx) was encapsulated for the lactate sensor. Both sensors demonstrated excellent electrocatalytic activities and the results correlated well with the assay kit analysis. This approach not only enhanced the electrochemical and catalytic properties of the enzymes but also enhanced operational and thermal stability and their reusability. These results corroborate that, encapsulating enzymes in the MOF structure improves stability and catalytic activity and retains conformational orientation while protecting the enzymes from harsh conditions such as varying pH and high temperatures. This development opens new pathways for the everyday utilization and industrial application of enzymes. The electroanalytical performances of various MOF-based wearable and portable electrochemical glucose sensors are displayed in Table 1.

### 2.5. Isopentane

A noninvasive and ultrasensitive electrochemical screening device for the early diagnosis of lung cancer through isopentane monitoring was fabricated using a 1-butyl-3-methylimidazolium tetrafluoroborate ionic liquid (BMIM-IL)-integrated ZIF-8 MOF [73]. The good conductivity of ILs enhances the electrochemical and electrocatalytic activity of the sensor, while the large active surface area of MOFs improves gas adsorption. The developed sensor quantified isopentane from 600 ppb with excellent sensitivity and selectivity. Moreover, this is the first report of a hydrocarbon-based sensing platform developed for the diagnosis of lung cancer. Using this sensor, a portable microelectronic prototype was constructed as a proof of concept, which successfully quantified isopentane concentrations. This sensor opens new pathways for the real-time monitoring of lung cancer, using isopentane as a biomarker.

### 2.6. Isoprene

Isoprene is a volatile organic compound released endogenously in the breath of a person affected by the influenza virus or respiratory inflammation. Therefore, isoprene monitoring is essential to identify and restrict the disease’s spread. The current breath-based analysis involves sophisticated instruments which require trained personnel, is time-consuming, and cannot be utilized in PoC applications. Banga et al. developed an electrochemical nose-based detection system for the sensing of isoprene using a GNP-encapsulated zinc MOF [74]. The developed nanocomposite was thoroughly characterized and deposited over a custom-made three-electrode system attached to a PCB, where the WE was made of nickel immersion gold on a copper track; the CE and RE were made of carbon and Ag/AgCl, respectively. Thus, the developed sensor quantified isoprene with high selectivity and sensitivity over an LOD of 10 ppb in air. Using this sensor, a PoC device was constructed for the remote determination of isoprene, successfully detecting isoprene in real-time.

### 2.7. Lactate

Lactate is an important cellular metabolite and the end-product of pyruvate during anaerobic glycolysis. It is primarily produced in red blood cells, the brain, muscles, gut, and skin. Lactate is a critical biomarker for exercise and physical activity, making it useful for evaluating the physical fitness of athletes. Additionally, elevated lactate levels can indicate various conditions such as sepsis, hypoxia, metabolic acidosis, skeletal muscle fatigue, myocardial infarction, and acute respiratory distress [75]. Therefore, the accurate detection and continuous monitoring of lactate are essential in sports medicine, clinical diagnostics, and the food industry. Recently, Chang and colleagues developed a lactate biosensor using nitrogen-doped graphene quantum dots (N-GQDs) embedded in NiCo-MOF-derived layered double hydroxides (LDHs) [76]. Initially, N-GQDs were synthesized separately and then incorporated during MOF synthesis. The resulting N-GQD@MOF was subjected to LDH formation via a hydrothermal reaction in KOH. The final product was deposited onto a screen-printed carbon electrode (SPCE) and utilized for lactate analysis in perspiration, demonstrating excellent electrocatalytic activities. The sensor exhibited a linear detection range of 0 to 15 mM, with a sensitivity of 62.63 µA mM^−1^ cm^−2^ and an LOD of 0.252 mM at an operating potential of 0.6 V.

### 2.8. Levodopa

**Figure 7:**
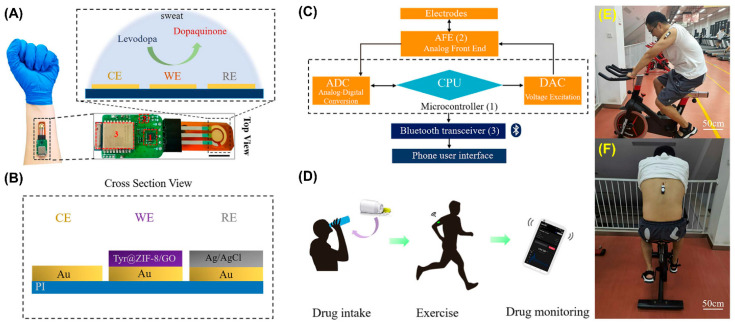
(**A**) Photograph of wearable LD sensor and its electrochemical sensing of LD. (**B**) Graphical representation of various components of the three-electrode system. (**C**) Block diagram of the potentiostat. (**D**–**F**) Real-time application of the fabricated LD sensor. Reproduced with permission from [77].

Levodopa (LD) is one of the most essential drugs used in the treatment of Parkinson’s disease. In the brain, LD is converted into dopamine, which aids in improving motor symptoms. However, excessive intake or overdose of LD can lead to lethal effects such as tardive dyskinesia. Therefore, it is essential to monitor the concentrations of LD levels in patients. Xiao and colleagues recently developed a wearable LD sensor using a tyrosinase-embedded ZIF-8/GO nanocomposite [77]. The nanocomposite was formed by introducing GO during the synthesis of the ZIF-8 network, and the tyrosinase enzyme was physically mixed with the nanocomposite to create the desired electrocatalyst. This composite was then coated over a screen-printed AuE attached to a portable potentiostat. The sensor demonstrated good selectivity and reproducibility, with a linear detection range of 1–95 µM and an LOD of 0.45 µM, respectively. Additionally, the sensor was applied to detect LD in sweat samples of human subjects with high LD levels. The results obtained from the subjects using the designed sensor were compared with those from an electrochemical workstation, showing an agreeable correlation. The improved performance of this sensor can be attributed to the incorporation of the enzyme into the MOF/GO composite, which efficiently preserved the enzyme’s nativity and enhanced the catalytic activity of the fabricated sensor. A schematic representation of the various components of the LD sensor and its practical applicability are displayed in Figure 7.

### 2.9. Metal Ions

Elashery and colleagues developed a flexible and wearable sensor using a 2D Ni-MOF NS-coated activated flexible porous carbon cloth decorated with nitrogen and carbon nanoparticles (AFPC-CNPs) to detect nickel ions in sweat droplets [78]. Nickel is an essential metal widely used in various applications, ranging from everyday stainless-steel products to batteries and aerospace equipment. Additionally, nickel is naturally present in soil, water, microorganisms, and plants. However, nickel exposure can significantly impact human health, leading to allergic reactions, respiratory issues, systemic toxicity, and carcinogenicity. In this study, the sensor was fabricated by synthesizing AFPC-CNP via the electropolymerization of pyrrole over cotton fabric, followed by carbonization at 850 °C and activation with KOH. Finally, the AFPC-CNP was dip-coated with separately synthesized Ni-MOF NSs to form the desired sensor. Initially, the performance of the sensor toward nickel ions was evaluated using a paste electrode and later, the applicability of the flexible sensor was investigated. The developed sensor exhibited a linear detection range of 10 µM to 0.1 M with an LOD of 2.7 µM. Furthermore, the fabricated sensor showed excellent selectivity for nickel ions, with good repeatability, accuracy, and intermediate precision. The sensor was also extended to analyze biological samples such as sweat, saliva, and environmental samples like tap water and pure water.

In another study, a multiplexed heavy metal ions sensor was developed using a bismuth–copper bimetallic MOF-derived carbon film, which encapsulated Bi-Cu alloy nanoparticles (BiCuANPs@CF) [79]. Incorporating bismuth with copper enhances electrocatalytic activity and acid resistance, while encapsulation within the CF improves electron transferability and mitigates volume change during the adsorption and desorption of metal ions. As a result, the developed hybrid exhibits excellent conductivity, high stability, and facile charge transferability, thus significantly improving the overall performance of the sensor. The sensor demonstrated broad linear detection ranges of 150–600, 5–900, and 0.5–700 ppb with low LODs of 35, 0.95, and 0.081 ppb for Zn^2+^, Cd^2+^, and Pb^2+^ ions, respectively. The applicability of the sensor in real samples was investigated using human biofluids and various water samples, which showed acceptable recoveries. Additionally, the sensor was integrated into a handheld portable device for the remote/onsite monitoring of heavy metal ions, demonstrating excellent applicability in PoC technology.

### 2.10. Melatonin

**Figure 8:**
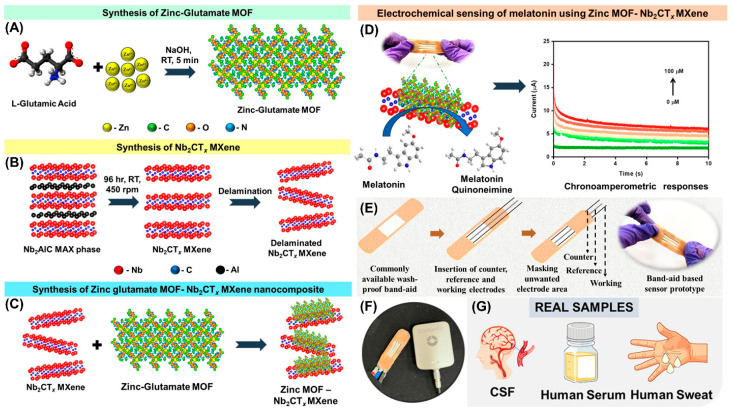
(**A**) Graphical representation for the synthesis of (**A**) zinc–glutamate MOF, (**B**) MXene, and (**C**) MOF-MXene nanocomposite. (**D**) Electrochemical sensing of melatonin and its amperometric response. (**E**) Scheme for the fabrication of band-aid based prototype melatonin sensor. (**F**) Photograph of prototype sensor connected to the portable potentiostat. (**G**) Various real samples analyzed. Reproduced with permission from [80].

A highly sensitive melatonin sensor was developed using an MOF-embedded MXene nanocomposite [80]. Melatonin, a sleep hormone, plays a significant role in regulating the sleep–wake cycle, also known as the circadian rhythm. Its production and release from the brain are connected to the time of day, increasing when it is dark and decreasing when it is light. Abnormal levels of melatonin can lead to various conditions, including sleep disorders, dementia, stress, hypertension, and Alzheimer’s disease. In this study, a carbon yarn coated with a zinc–glutamate MOF embedded in a Nb_2_CT_x_ MXene nanocomposite was prepared and utilized as a sensor for the sensitive detection of melatonin. This novel sensor exhibited a linear detection range from 1 to 100 µM with an LOD of 215 nM. The superior performance of the sensor could be attributed to the inclusion of the bio-MOF and MXene in the sensor, which synergistically improved the electrocatalytic properties, increased active sites, and enhanced electrical conductivity. The developed sensor also detected melatonin in biofluids such as cerebrospinal fluid, sweat, and blood serum. The sensor was further adapted into a wearable band-aid design, incorporating an MOF-MXene-coated carbon yarn as the working electrode, a Ag/AgCl wire as the RE and an unmodified carbon yarn as the CE, as illustrated in Figure 8. This flexible band-aid sensor was connected to a portable potentiostat, which successfully performed the electrochemical analysis of melatonin.

### 2.11. Nerve Agent

Nerve agents (NAs) are a class of organic compounds that disrupt the mechanism by which nerves transmit messages to organs. Additionally, they are extremely toxic and potentially fatal. NAs function by inhibiting the enzyme acetylcholinesterase, which is responsible for breaking down acetylcholine [81]. Therefore, the onsite detection and remote monitoring of NAs are crucial for health and environmental protection. Sandhu and colleagues developed a wearable potentiometric sensor for the detection and degradation of NAs using a biomimetic Zr-MOF (MIP-202(Zr)) [82]. The sensor consists of a biocompatible and thermally stable MIP-202(Zr) coupled with a fluoride ion-selective electrode (FISE) transducer, which was utilized for sensing diisopropylfluorophosphate (DFP), an F-containing G-type NA simulant. The Zr-MOF was synthesized by a hydrothermal reaction between ZrCl_4_, and L-aspartic acid and the resulting MOF crystals were activated using Soxhlet extraction and stored in a vacuum. The working electrode was made by dispersing the Zr-MOF with PVDF, which was then coated over the FISE. The developed MIP-202 sensor efficiently degraded DFP at a near-neutral pH and demonstrated excellent thermal and storage stability (up to 30 days at 60 °C). Furthermore, the sensor was adapted into a wearable textile-based format and utilized for the real-time analysis of DFP aerosols.

### 2.12. Sulfur Mustard Stimulant

**Figure 9:**
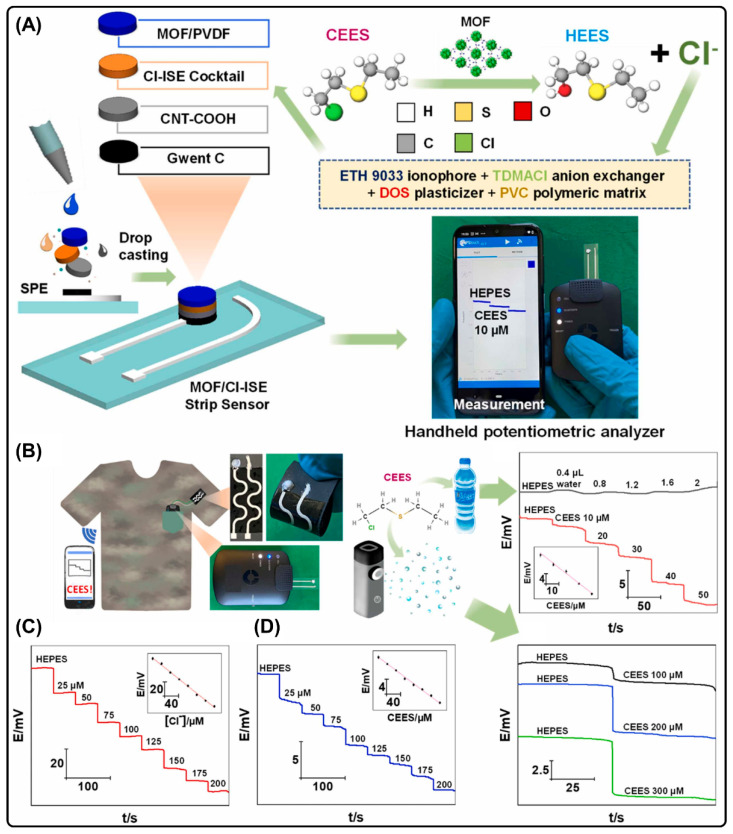
(**A**) Schematic illustration of various components of the sensor and CEES sensing mechanism. (**B**) Wearable and PoC sensor and potentiometric responses obtained for drinking water and aerosols. (**C**,**D**) Potentiometric response obtained for textile-based wearable sensor for various concentrations of Cl ions and CEES. Reproduced with permission from [83].

Sulfur mustard is a chemical warfare agent that was first introduced in WWI and recently during the Iran–Iraq war. In order to protect civilians, homeland security, and the environment, it must be detected promptly to enable timely and efficient countermeasures. Sandu et al. developed a solid-contact chloride ion-selective (Cl-ISE) potentiometric sensor for the detection of 2-chloroethyl ethyl sulfide (CEES), a sulfur mustard stimulant [83]. The sensor was fabricated by coating a biomimetic zirconium MOF (MIP-202) mixed with PVDF over a Cl-ISE and CNT-modified carbon strip electrode (Figure 9). The developed sensor successfully quantified CEES in multiphasic samples, including buffer solutions, drinking water, and buffered aerosols. Furthermore, the sensor was adapted for handheld portable sensing systems and textile-based wearable fabrics, which exhibited acceptable performance.

### 2.13. Sweat Monitoring

**Figure 10:**
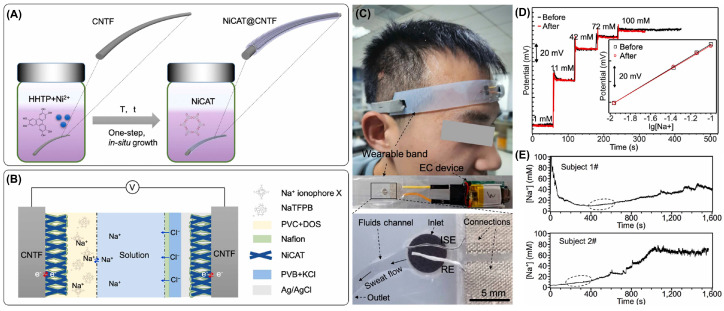
(**A**) Scheme for the synthesis of MOF@CNTF and (**B**) electrochemical cell. (**C**) Photograph of a volunteer wearing the band-based wearable sensor and its components. (**D**) Potential response and (**E**) real-time sweat sodium concentrations of two subjects obtained using the wearable sensor. Reproduced with permission from [85].

Sweat monitoring or perspiration analysis is highly significant, as it contains various essential biomarkers related to several biological processes taking place in the body. Therefore, continuous or periodic monitoring could give multifarious health information that can aid in the early diagnosis of various diseases, fitness monitoring, sports medicine, and so forth. A multifunctional patch for the real-time sensing of sweat electrolytes (Na^+^, K^+^, Ca^2+^ ions, and pH) was developed using a nanocomposite consisting of MWCNT and MOF-derived core–shell nanoporous carbon (CSNPC) [84]. The CSNPC was synthesized from the Co and Zn MOF structures via solvothermal, carbonization, and etching processes. CSNPC@MWCNT was prepared by ultrasonically mixing both compounds in a 3:1 ratio, yielding the desired electrocatalyst. The sensor was fabricated by the laser etching of polyimide films and then coated with the synthesized CSNPC@MWCNT catalyst. After that, the sensors were coated with respective ion-selective membranes made of different ratios of sodium tetrakis [3,5-bis(trifluoromethyl)phenyl] borate, polyvinyl chloride, bis(2-ethylehexyl)sebacate, sodium tetraphenyl borate, tetrahydrofuran, calcium ionophore II, valinomycin, and PANI. The detailed fabrication protocols are given in the literature [84]. Finally, the sensor was attached to a microfluidic sweat collection channel and microcontroller to monitor the real-time analysis of perspiration. The developed patch sensor successfully quantified various target ions present in human sweat. Another sweat sensor was developed for the potentiometric sensing of sodium ions using MOF and CNT fibers coupled with a solid-contact ion-selective electrode [85]. Following a one-step hydrothermal method, a vertically aligned nickel triphenylene-fused metal catecholate MOF was synthesized on CNT fibers, and then Nafion was coated to improve the sensors’ hydrophobicity. The resulting Nf/MOF@CNTF was modified with a sodium ion-selective membrane to obtain the working electrode, while the reference electrode was obtained by modifying the Nf/MOF@CNTF with Ag/AgCl ink and a polyvinyl butyral resin solution containing KCl. Due to its ordered porous architecture, the developed sensor exhibited enhanced double-layer capacitance and low contact impedance. A wearable band-based prototype was fabricated using this sensor, which successfully quantified the sodium ions present in the volunteers’ sweat during exercise (Figure 10).

### 2.14. Uric Acid

**Figure 11:**
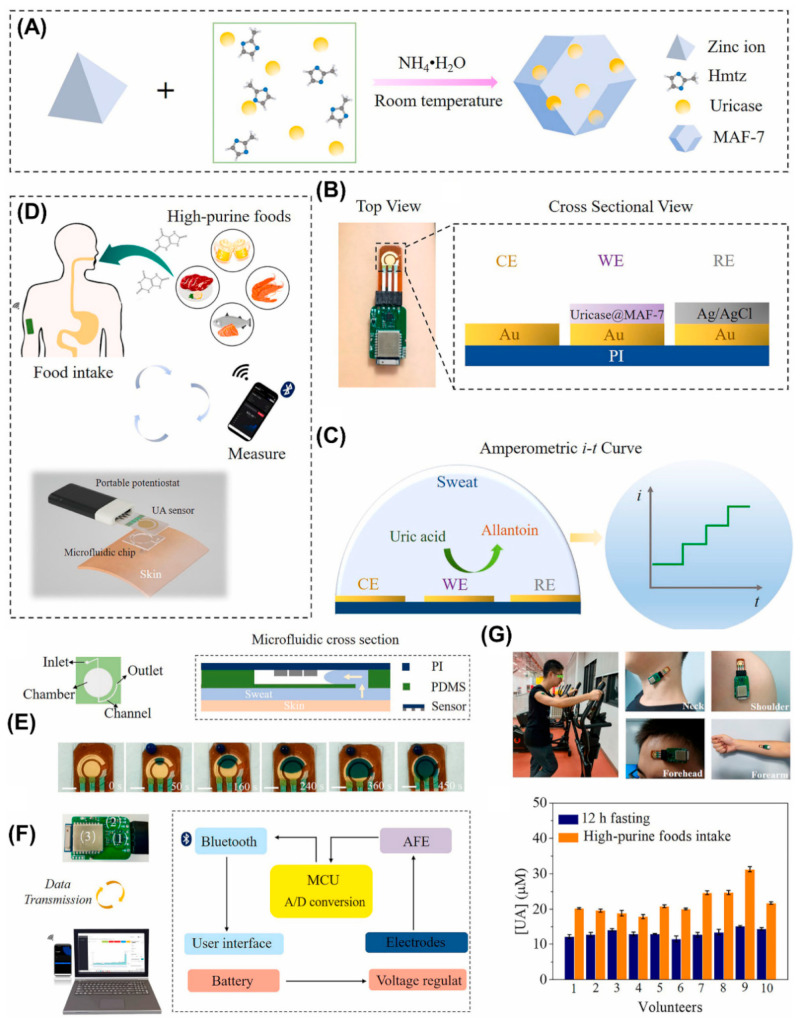
(**A**) Scheme for the synthesis of UC@MAF7. (**B**) Top view and cross-sectional view of the fabricated sensor. (**C**) UA sensing mechanism. (**D**) Schematic illustration of sensor fabrication and sensing mechanism. (**E**) Schematic representation of top and cross-sectional view of the microfluidic channel and actual photographs of fluids flowing in the microfluidic channel at different time intervals. (**F**) Photograph of printed circuit board consisting of (1) microcontroller, (2) analog front end, and (3) Bluetooth transceiver along with block diagram of the PCB. (**G**) Photographs of the subjects wearing the sensor on different parts of the body and UA concentrations measured in 10 volunteers. Reproduced with permission from [86].

Uric acid (UA) is a critical biomarker for various physiological and pathological conditions, making its detection and monitoring essential in clinical diagnostics, healthcare, and wellness management. Xiao and colleagues developed a wearable, sweat-based UA sensor using uricase encapsulated within a hydrophilic zinc MOF (UC@MAF-7) [86]. During the synthesis of MAF-7, uricase was encapsulated within the MOF structure, which helped preserve the native structure of the uricase under harsh conditions and enhanced its enzymatic activity. The sensor was constructed by drop-coating the synthesized UC@MAF-7 onto a screen-printed gold three-electrode setup. Initially, the sensor’s applicability was evaluated in artificial sweat and later extended to human sweat analysis. This sensor demonstrated a linear detection range of 2–70 µM with an LOD of 0.34 µM for UA detection. For human subject analysis, the sensor was attached to a flexible microfluidic channel placed on the skin of the subject. The microfluidic channel effectively collected the sweat and delivered it to the sensor surface. A portable potentiostat attached to the sensor performed the measurements and displayed the quantification data on a smartphone screen. The sensor fabrication, sensing mechanism, various components of the fabricated sensor, and human trials are shown in Figure 11.

## 3. Challenges in MOF-Based Sensing Devices

Despite the significant advantages of MOFs, several challenges must be addressed before they can be fully utilized in wearable, flexible, and PoC sensors. These challenges can be broadly classified into property-oriented and applicability-oriented categories. Key properties of MOFs that require optimization include stability, conductivity, biocompatibility, and selectivity, which are all interconnected and directly influence the performance of fabricated sensing devices. Improving the stability and selectivity of MOFs is crucial, as these factors impact the practical utility of sensors in complex real-world samples. Enhancing conductivity is also essential for developing ultrasensitive sensors, as it significantly affects the sensitivity and detection limits of the fabricated sensors. These properties can be improved by strategically tuning the functional groups in the organic linkers, doping nanostructures during or after synthesis, entrapping/encapsulating different functional moieties within the porous structures, or forming composites with other materials and molecules. Another challenge is that most synthesized MOFs are produced in powder form, which limits their direct application in wearable and flexible sensors. To address this, MOFs need to be processed into thin films for device integration. This can be achieved by mixing MOFs with support materials (such as polymers or carbon nanomaterials) or by shaping or printing them with substrate materials. Common techniques for integrating MOFs into devices include in situ growth, spin/spray coating, drop-casting, electrodeposition, atomic layer deposition, layer-by-layer growth, and various printing methods (3D, screen, or inkjet printing). Each technique has its own advantages and limitations, and the most suitable one should be selected based on the application and nature of the sensing device. Additionally, most MOF-based sensors developed to date are screening-based, where the target analyte is identified by testing a range of possible analytes. To design more target-specific MOFs, further research into experimental methods and computational simulations is required.

## 4. Summary and Future Prospects

Healthcare is becoming one of the most fundamental human necessities due to the widespread prevalence of infectious and chronic diseases. Diagnosing and treating billions of people is both time-consuming and costly. Moreover, most current diagnostic methods are invasive, potentially causing injuries and infections. These methods also often require expensive and bulky equipment, skilled operators, and are typically only available in large facilities, limiting their accessibility for immediate needs. The advent of flexible and portable electronics has brought significant attention to wearable and PoC sensors, which are instantaneous, noninvasive, cost-effective, and capable of remote and continuous monitoring. In this context, electrochemical-based wearable and PoC sensors are highly favored because of their advantages, such as high sensitivity, selectivity, rapid response times, portability, and tailor-made properties. Among the various electrode materials explored, metal–organic frameworks (MOFs) are among the most suitable candidates due to their highly porous crystalline structure, large surface area, customizable surface functionalities, and tunable porosity, which can be sustainably synthesized in large quantities. Furthermore, MOFs exhibit fascinating properties that make them particularly interesting for electrochemical applications, including energy generation, storage, and electrochemical sensing. Owing to these advantages, several MOF-based electrochemical sensors and biosensors have been developed recently. However, the field of MOF-based wearable sensors is still emerging, with few reports available in the literature. Therefore, it is an opportune moment to write a focused review highlighting the significance of MOF-based wearable sensors for healthcare monitoring. The large active surface area of MOFs enhances the adsorption of reactants, facilitating their detection and conversion. The tunable porosity of MOFs can be strategically utilized to trap or encapsulate various biomolecules and redox species, enhancing the selectivity, stability, and activity of the designed sensors. Additionally, chemical functionalities can be engineered on the surfaces or even within the pores of MOFs, improving the specificity and catalytic activity of the sensors. Furthermore, the sustainable synthesis of MOFs in large quantities could reduce both the cost and environmental impact of these sensors, making them affordable for widespread use. Despite the promising performance of MOF-based electrochemical sensors, several challenges need to be addressed for the successful integration of MOF-based wearable sensors. Many of the limitations of pristine MOFs can be mitigated by forming composites and hybrids with other nanomaterials and functional molecules. Although several MOF-based electrochemical sensors exist, few have been integrated into wearable and portable devices. Therefore, considerable efforts are needed to advance this field, including improving the stability, sensitivity, and lifespan of the sensing systems, preventing microbial growth on sensor surfaces, and ensuring easy recovery and disposal after use. These steps are essential to ensure that the proposed wearable sensing systems can perform their intended functions effectively in real-world scenarios.

**Figure 12:**
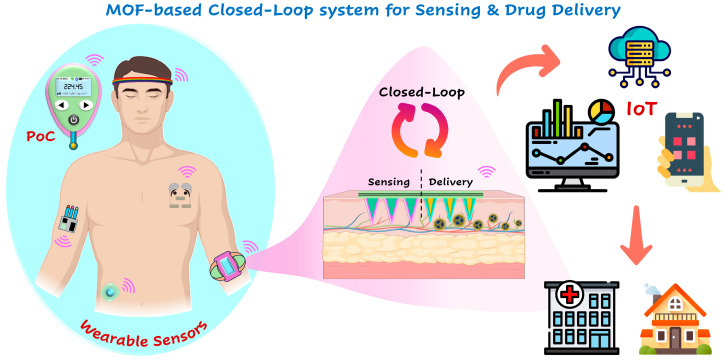
Graphical illustration of proposed closed-loop sensing and drug delivery system using MOF-based electrochemical sensors. (A part of this image was designed using icons from www.flaticon.com, accessed on 25 August 2024).

Furthermore, by leveraging the large surface area, porous structure, and high loading capacity of MOFs, drugs can be stored and released in an on-demand manner, similar to advanced drug delivery systems. By incorporating these strategies, the sensing platform can also facilitate targeted drug release. Integrating algorithms allows wearable devices to quantify bio-analytes in real-time and autonomously release the required amounts of drugs as a countermeasure without human intervention. This approach opens new avenues for developing closed-loop systems that can simultaneously or periodically measure bio-analytes and release drugs as needed. Integrating these sensors with digital health platforms and the IoT could enable real-time data analysis, promoting a proactive approach to health management, disease prevention, and treatment, as these devices can effectively track patient health and communicate with patients and healthcare professionals (Figure 12). Therefore, MOFs provide a promising platform for designing and developing advanced sensing and drug delivery systems. Ongoing research is expected to address current limitations and pave the way for the widespread use of MOFs in sensing and on-demand drug delivery. As the field continues to evolve, MOFs have the potential to revolutionize how drugs are monitored and delivered, enhancing the efficacy and safety of diagnostics and treatments for various diseases and disorders. Addressing the challenges of developing and implementing these technologies strengthens the potential to make personalized medicine a reality, ushering in a new era of healthcare monitoring.

## Figures and Tables

**Table 1:** Electroanalytical performances of various MOF-based wearable and portable glucose sensors.

Sensors	Linear Range (µM)	LOD (µM)	Sensitivity	pH	Potential (V)	Ref.
PVA/KOH/NiCo-MOF/GCE	5–205; 205–2655; 2655–5655	0.11	1422.2; 522.9; 285.8 µA mM^−1^ cm^−2^	Basic	0.50	[63]
PVA/KOH/NiCo-MOF/PET	10–200	10	0.31 µA µM^−1^			
NiCo-MOF/Ag/RGO/PU	10–660	3.28	425.9 µA mM^−1^ cm^−2^	7.0	0.50	[64]
NiCo-MOF/NF	40–5340	1.68	2.935 µA mM^−1^	Basic	0.60	[65]
Nf/NiCo-MOF/CNT/PDMS	20–1100	6.78	71.62 µA mM^−1^ cm^−2^	Basic	0.50	[66]
	20–280	-	-	7.4		
ZIF-8/RGO/PGE	5–5000	0.3	5047.18 µA mM^−1^ cm^−2^	7.4	0.35	[67]
Pd@Co-MOF/PET	10–1000	2.0	-	-	0.60	[68]
Cu-MOF/PtNPs/AuE	400–25,000	60	158.41 µA mM^−1^ cm^−2^	Basic	0.55	[69]
GOx/Hemin@NC-ZIF	50–600	2.0	-	7.2	0.60	[70]
PSM/GOx/PB/HNPC/PIF	0–1500	0.025	82.7 µA mM^−1^ cm^−2^	7.4	−0.05	[71]
GOx-CDs@ZIF-8	0–7000	12.5	9.28 µA mM^−1^ cm^−2^	Basic	−0.05	[72]

## Data Availability

No data were used for the research described in the article.

## Abbreviations

AuE	Gold electrode
BMIM	Butylmethyl imidazolium
CC	Conductive carbon
CD	Carbon dots
CE	Counter electrode
Cl-ISE	Chloride-ion selective electrode
CNT	Chloride-ion selective electrode
CNTF	Carbon nanotube fiber
CPE	Carbon paste electrode
CRT	Creatinine
CSNPC	Core–shell nanoporous carbon
CuNPs	Copper nanoparticles
FISE	Fluoride ion-selective electrode
GO	Graphene oxide
GOx	Glucose oxidase
HNPC	Hybridized nanoporous carbon
IL	Ionic liquid
KOH	Potassium hydroxide
LD	Levodopa
LDHs	Layered double hydroxides
LOD	Limit of detection
LOx	Lactate oxidase
MOF	Metal–organic framework
MWCNT	Multiwalled carbon nanotube
NC	Nanocage
Nf	Nafion
N-GQDs	Nitrogen-doped graphene quantum dots
NiNPs	Nickel nanoparticles
PB	Prussian blue
PD	Parkinson’s disease
PDMS	Polydimethylsiloxane
PET	Polyethylene terephthalate
PGE	Pencil graphite electrode
PIF	Polyimide film
PoC	Point of care
PSM	Permselective membrane
PtNPs	Platinum nanoparticles
PU	Polyurethane
PVA	Polyvinyl alcohol
RE	Reference electrode
RGO	Reduced graphene oxide
SPCE	Screen-printed carbon electrode
UA	Uric acid
WE	Working electrode
ZIF	Zeolitic imidazolate frameworks
